# Supplementary material for: A Critical View of the Application of the APEX Software (Aqueous Photochemistry of Environmentally-Occurring Xenobiotics) to Predict Photoreaction Kinetics in Surface Freshwaters
Source: Molecules. 2019 Dec 18;25(1):9. doi: 10.3390/molecules25010009 (PMC7017383; doi:10.3390/molecules25010009)
Supplement: Supplementary file 1 [file molecules-25-00009-s001.zip › 3.2.4_gcc-4.4.0.zip!/3.2.4_gcc-4.4.0/3.2.4_gcc-4.4.0/doc/octave/html/interpreter/Escape-Sequences-in-string-constants.html]

Escape Sequences in string constants - Untitled


Next: Character Arrays,
Up: Strings


---

### 5.1 Escape Sequences in string constants

In double-quoted strings, the backslash character is used to introduce
escape sequences that represent other characters. For example,
\n embeds a newline character in a double-quoted string and
\" embeds a double quote character. In single-quoted strings, backslash
is not a special character. Here is an example showing the difference:

```
     toascii ("\n")
         => 10
     toascii ('\n')
         => [ 92 110 ]
```

Here is a table of all the escape sequences used in Octave (within
double quoted strings). They are the same as those used in the C
programming language.

`\\`: Represents a literal backslash, \. `\"`: Represents a literal double-quote character, ". `\'`: Represents a literal single-quote character, '. `\0`: Represents the “nul” character, control-@, ASCII code 0. `\a`: Represents the “alert” character, control-g, ASCII code 7. `\b`: Represents a backspace, control-h, ASCII code 8. `\f`: Represents a formfeed, control-l, ASCII code 12. `\n`: Represents a newline, control-j, ASCII code 10. `\r`: Represents a carriage return, control-m, ASCII code 13. `\t`: Represents a horizontal tab, control-i, ASCII code 9. `\v`: Represents a vertical tab, control-k, ASCII code 11.

In a single-quoted string there is only one escape sequence: you may insert a
single quote character using two single quote characters in succession. For
example,

```
     'I can''t escape'
         => I can't escape
```
